# The prevalence of painful diabetic neuropathy in 300 Moroccan diabetics

**DOI:** 10.11604/pamj.2018.31.158.14687

**Published:** 2018-11-01

**Authors:** Zakaria Chahbi, Bouchra Lahmar, Sanae El Hadri, Lahoussaine Abainou, Said Kaddouri, Hassan Qacif, Hicham Baizri, Mohamed Zyani

**Affiliations:** 1 Internal Medicine Department, Avicenne Military Hospital, Marrakech, Morocco; 2 Endocrinology Department, Avicenne Military Hospital, Marrakech, Morocco

**Keywords:** Diabetes, DN4 questionnaire, neuropathic pain, prevalence

## Abstract

Painful diabetic neuropathy is a frequent complication of diabetes. Its diagnosis is clinical. Our goal is to determine the prevalence of painful diabetic neuropathy in this population. We also analyzed the relationship between this neuropathy and certain parameters, concerning the patient and his diabetes. It is a cross sectional study conducted at the department of endocrinology and internal medicine of Avicenne hospital Marrakech-Morocco, among a cohort of 300 diabetic outpatients. We used the DN4 questionnaire (Douleur Neuropathique en 4 questions), for diagnosis. The results showed a prevalence of 15%. In this study: advanced age, female gender, duration of diabetes greater than 10 years, and the lack of medical follow up were found to be statistically significant risk factors for painful diabetic neuropathy, in addition to some diabetes-related comorbidities such as hypertension, dyslipidemia, sedentary life style and diabetic retinopathy. Painful diabetic neuropathy remains undertreated, in fact 74% of our patients did not receive any specific treatment, knowing that the progress in developing effective and well-tolerated therapies has been disappointing.

## Introduction

Diabetes mellitus is one of the biggest global health problems of the 21^st^ century. Its global prevalence in adults aged 20-79 years was estimated to be 8.8% [1]. In Morocco, it was estimated to be 7.7% [1]. Diabetes could lead to serious complications; in this study we were interested in the painful form of peripheral neuropathy. The prevalence of painful diabetic neuropathy (PDN) varies between 10% and 60% [2]. Patients should be systematically questioned concerning suggestive symptoms as they do not spontaneously report them. PDN can have debilitating consequences with significant impact on the quality of life and cost of care. It is a complication that affects small fibers, thus it may be accompanied by a normal monofilament test and electromyogram (EMG) [3]. The diagnosis is clinical, based on clinical finding, and has been facilitated by the development of simple and validated questionnaires, such as the neuropathic pain 4 questions (DN4) [4]. To date, glycemic control remains the most effective way to slow down and prevent PDN. In this study, we have determined the prevalence of PDN and its potential risk factors.

## Methods

It is an observational, cross-sectional study, including 300 Moroccan diabetic out-patients, recruited from the departments of internal medicine and endocrinology departments, in Avicenne Hospital in Marrakech. A score of ≥ 4 on the DN4 was used to establish the diag-nosis of PDN (Annex 1). The exclusion criteria were as follows: duration of type 1 diabetes mellitus less than 5 years, the presence of a psychiatric disorder that could influence the reliability of the questionnaire, patients with lower limb amputation, the presence of any other type cause of neuropathic pain (such as: post-herpetic neuralgia, pain associated with cancer, pain related to spinal cord injury, multiple sclerosis), being under certain drugs witch can give peripheral neuropathy (antiretroviral therapy , cisplatin, thalidomide, metronidazole). The statistical analysis was performed using SPSS V.20. Variable were compared using the χ^2^ and Fisher exact tests and p-value <0.05 was con-sidered to be statistically significant.

## Results

**Figure 1:**
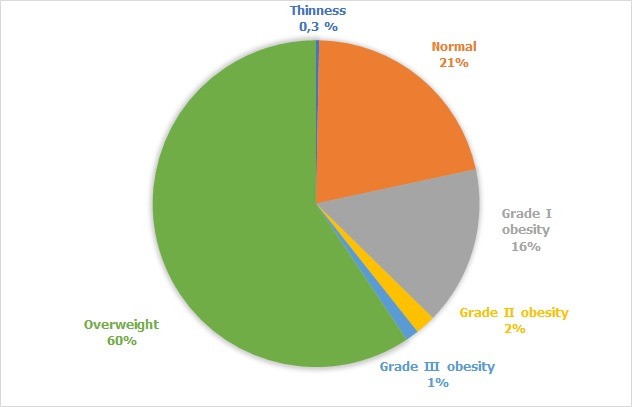
Patients distribution according to body mass index (N = 300)

**Figure 2:**
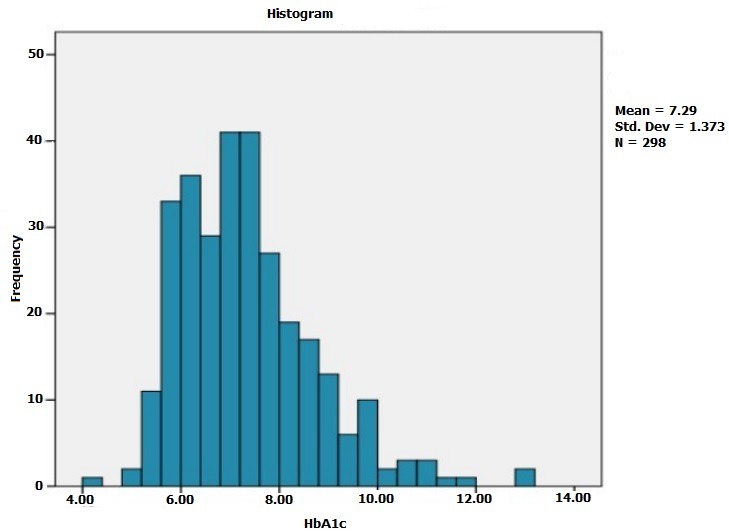
Distribution of HbA1c values in our cohort (N = 298)

**Figure 3:**
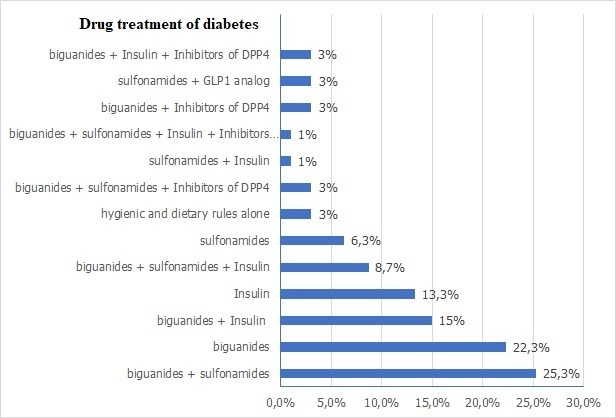
Drug treatment of diabetes

**Figure 4:**
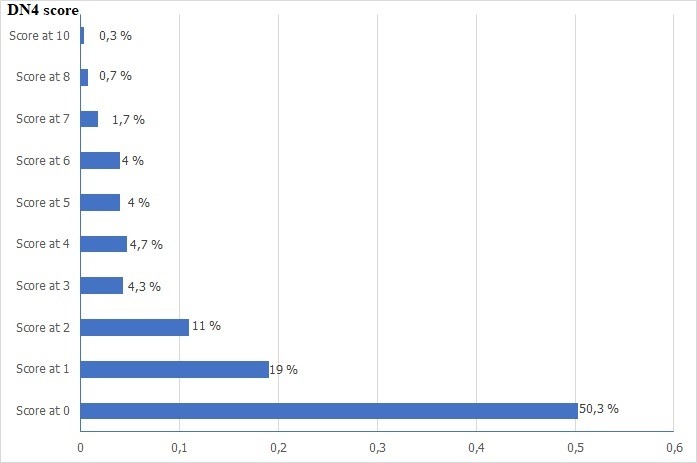
Results of the DN4 score

The mean age of patients enrolled in the study was 57.24 ± 9.79 years [32-85 years]. Males were predominant with a sex ratio (F/M) = 0.93. The men body mass index (BMI) was 27.53 ± 3.67 Kg / m^2^, 59.3% of these patients were overweight and 18% were obese (Figure 1). The majority of the patients had Type 2 diabetes (95.7%), with a mean duration of 10.63 ± 7.47 years. Also 95.7% of the patients were followed-up for their diabetes. The mean glycosylated hemoglobin (HbA1c) of the last 3 months was 7.29% ± 1.37% (Figure 2). The treatment received by the patients for diabetes manage-ment is summarized on Figure 3. Concerning cardiovascular risk factors: 44.3% of the patients had hypertension, while 11.7% had no known blood pressure status. Dyslipidemia was found in 46.3% of cases. Only 22.7% reported a sedentary lifestyle. 12% of the patients were current smokers or had quit less than 3 years at the moment of the study, with a mean of 24.86 ± 11.4 pack-year smoking history. Only 3 patients reported a chronic alcohol consumption. Among diabetes complications, retinopathy was the most frequent (34.3%), dominated by stage I of the world health organization (WHO). Nephropathy was noted in 26.3% of cas-es, dominated by stage III. A history of coronary artery disease (CAD) was found in 11% of patients, followed by peripheral artery disease (PAD) and ischemic stroke in 2.7% of cases each. In this cohort, 15.4% (95% CI 11-19) of the patients had a DN4 score ≥ 4 and met the diagnostic criteria of PDN. The mean DN4 score was 1.37 ± 1.98 (Figure 4). Burning (23.7%), and tingling (23.7%) were the most common symptoms and were reported equally, followed by numbness (23.3%) and electric shocks (15.7%).

In the study of the relationship between PDN and certain parameters, concerning the patient and his diabetes, advanced age was significantly associated with the presence of PDN (p = 0.017). In fact, a linear relationship was observed between PDN and increasing age (Table 1). Also, female gender (p=0.03), duration of diabetes >10 years (p = 0.019) and the absence of diabetes follow-up (p<0.0001) were all associated with the presence of PDN (Table 2). Although obesity (BMI>30) and poor glycemic control (HbA1c >7%) were found to be associated with PDN, the data were not statistically significant. Almost all of our patients had type 2 diabetes, as such, a relationship between the type of diabetes and PDN couldn't be established in this study. Cardiovascular risk factors that were found to be significantly associated with PDN are: hypertension (p=0.001), dyslipidemia (p <0.0001) and sedentary life style (p <0.0001). Chronic alcoholism and smoking didn't show any association. For degenerative complications, retinopathy was found to have a strong association with the presence of PDN (p <0.001). While nephropathy, PAD, CAD and a history of ischemic stroke showed no association to PDN in this study. In total, only 26% of our patients were treated for neuropathic pain, different medication used is presented on (Table 3).

**Table 1:** Frequency of painful diabetic neuropathy as a function of age (N = 300)

Age groups	Painful diabetic neuropathy	
	No**	Yes
<50	52(89.7)	6(10.3)
50-64	151(87.3)	22(12.7)
≥65	51(73.9)	18(26.1)
Total	254(84.7)	46(15.3)

P-Value= 0.017 S**

**Table 2:** Frequency of painful diabetic neuropathy by age of diabetes (N = 300)

Age of diabetes	Painful diabetic neuropathy N(%)		Total	P-Value
	No	Yes		
**< 10 years**	147(57.9)	18(39.1)	165(55)	0.019 S**
**≥ 10 years**	107(42.1)	28(60.9)	135(45)	
**Total**	254(100.0)	46(100.0)	300(100.0)	

**Table 3:** Therapeutic classes used by our cohort and their frequency

Therapeutic classes		Frequency
**Anticonvulsants**	Pregabalin	1
**Tricyclic aintidepressants**	Amitriptyline	4
**Inhibitor of serotonin reuptake**	Duloxetine	1
	Fluoxetine	1
**Analgesics Opioids**	Tramadol	2
**Associations**	Amitriptyline + group B vitamin	1
	Gabapentin + Paroxetine	1
	Pregabalin + Amitriptyline	1
**Total**		12

## Discussion

Diabetic neuropathy is one of the most common complications of diabetes, affecting nearly half of diabetics [5], it is often asymptomatic. It is made of many syndromes; the most common form is Distal symmetric sensorimotor polyneuropathy (DSPN) [2]. Neuropathic pain is defined as pain arising as direct consequence of a lesion or disease affecting the somatosensory system [6]. The origin of pain in the PDN is not fully elucidated. Chronic hyperglycemia seems to play a key role in it, as it induces ionic dysregulation and hypoxia in peripheral neurons, particularly at the level of nociceptors [3]. It could also to excessive production of certain metabolites, such as methyl glyoxal which increases the excitability of nociceptive receptors [7]. Such hyperexcitability is also described at the level of the spinal ganglion, which constitutes a therapeutic target for pregabalin and gabapentin. Neuropathic pain can also occur acutely, following a rapid glycemic control, more often following an insulin therapy, referred to as insulin neuritis [3]. Other accessory mechanisms that could explain neuropathic pain is the role of sympathetic system in the sensitization and inhibition of pain [8]. This complexity of PDN pathophysiology offers a multitude levels of therapeutic interventions the future. Epidemiological data on PDN are rare, as few studies have focused solely on this form of diabetic neuropathy. The populations studied and the diagnostic tools are also highly variable. As a matter of fact, the prevalence of PDN is 8% in France [9]. While it is 53.7% in the Middle East [10]. In our study, the overall prevalence of PDN was 15.3%. This prevalence is in accordance with those found in the literature. It is closer to the Western series than to the Middle East. A clear predominance of PDN is observed in type 2 diabetes [2, 10]. In our study, no case of PDN was observed in type 1 diabetics which accounted for only 3.3% of the studied population. In the literature about 5% of patients with type 1 diabetes are affected by neuropathic pain.

There is little data on risk factors and co-morbidities related to PDN. In the literature, the link with advanced age is clearly established by several studies [10-12], including ours. In the present study the mean age of patients with neuropathic was 60.93 ± 9.76 years, moreover, we noted a statistically significant linear trend between increasing age and the prevalence of PDN. We found a significant higher prevalence of PDN in females compared to males with a F/M ratio close to 2, similar to other studies [10 ,12, 13]. Diabetes duration is a major and well-established risk factor of DSPN, regardless of the patient's age. A strong association have been reported between PDN and the duration of diabetes, especially after 10 years of evolution [10, 11]. In the present study, 60.9% the patients PDN had diabetes duration >10 years. Hyperglycemia is another major risk factor of peripheral neuropathy [14], consequently a HbA1c> 6.5% increases its risk by 5 folds [15]. Therefore, a strict control of blood glucose might slow down or prevent the progression of PDN [16]. In the present study, the diagnosis of PDN was associated with a poor glycemic control: 63% of patients with PDN had a HbA1c> 7%, however, these findings were not statistically significant. Comparable to other studies, Hypertension was significantly associated with PDN, 70% of patients with a DN4 score >4 had hypertension [17, 18]. Similarly, this study found a significant association between PDN and dyslipidemia, also demonstrated in other papers [19, 20]. Sedentary life style was also found to be a risk factor of PDN in our study, similar results were reported by Sui-Whi et al [14]. Obesity has been documented as a risk factor for PDN [2, 10, 11]. Our study has noted a positive correlation between the two, however it wasn't statistically significant. Similar to other studies, we showed no significant association between PDN and Smoking or alcohol consumption [21]. In general, it may be difficult to differentiate between DSPN with alcohol as a risk factor and alcoholic neuropathy, in a person with diabetes [21].

Among comorbidities closely associated with PDN, is diabetic nephropathy, which can be more frequent in the presence of DSPN [22]. Conversely, patients with diabetic nephropathy may exhibit more pronounced DSPN, with an increased risk of severe diabetic foot lesions [23]. Diabetic nephropathy as an independent risk factor for PDN, has not been demonstrated in our study [10]. As for nephropathy, retinopathy can be considered as a co-morbidity of PDN, our study found a significant association (p=0.001), in accordance with other studies [24]. Some studies have reported PAD as a risk factor for PDN, Ziegler et al found this risk to be multiplied by 9 [25-27]. However, we did not find such correlation with PAD, nor with coronary artery disease, in contrast with Jambart et al., who reported a significant association between a history of myocardial infarction and PDN [10]. The diagnosis of PDN is clinical. The semiology of neuropathic pain includes functional signs, such as painful or painless abnormal sensations (paresthesia, dysesthesia), with other phenomena resulting in the loss of the nerve function (sensory-motor deficit). The pain is often spontaneous and nocturnal. The commonly reported paresthesia is tingling, pins and needles, and numbness [28]. The questionnaires used for diagnosis of PDN are validated, simple and easy to use, they also have an excellent sensitivity and specificity [29, 30]. The DN4 questionnaire is composed of a set of 4 questions (two for patient's interview and two for physical examination) [31]. The test is considered positive for a score ≥4/10. Its sensitivity and specificity are excellent (82.9% and 89.9% respectively) [4]. In our study, we used the validated Moroccan Arabic version of the DN4-interview [32]. The physical examination should look for signs typically suggesting small fibers involvement: hypoesthesia to pinprick or/and to cold-warm, allodynia to touch or friction, or hyperalgesia. The monofilament test, tuning fork and the EMG, are only able to detect abnormalities affecting large fibers, thus they be strictly normal. EMG is then required for PDN. The intensity of pain should be measured once the diagnosis of PDN is established, using pain scales such as the visual analogue scale (VAS), in order to assess the response to treatment [4].

The PDN represents a therapeutic challenge both for the physician and the patient. In the present study, 74% of the patients were not receiving any specific treatment for their PDN. In the literature, 39% of the patients suffering from PDN are not treated [33]. The goal of the treatment is to reduce the pain and improve the quality of life. Treatment of PDN is based on three major approaches: intensive glycemic control and risk factor management, treatments based on pathogenetic mechanisms such as alpha-lipoic acid and aldose reductase inhibitors [34, 35], and symptomatic pain management, aiming for a relief of 30% to 50% of the symptoms. Most guidelines suggest to use as a first-line treatment: tricyclic antidepressant, serotonin reuptake inhibitor, or GABA analogues (gabapentin, pregabalin) [36] (Table 4). The initial choice may also be influenced by the associated comorbidities (depression, insomnia). In case of therapeutic failure (improvement <30%) with the recommended maximum dose, then changing the drug class is recommended. If the efficacy is > 30%, and the pain remains > 3/10, it is recommended to add another therapeutic class. As a third line treatment tramadol or even morphine could be used. Other therapies are under study. ABT-594 was effective but poorly tolerated [37]. Intradermal injection of botulinum toxin A was reported to improve the pain and the quality of life [38].

**Table 4:** Examples of advantages and inconveniences that may influence the choice of molecules most often used in painful neuropathy

INN	Trade name	Advantages	Inconveniences
Gabapentin	Neurontin* or generic	No major drug interactions	Side effects Titration. Cost3 times a day formulation
Duloxetin	Cymbalta*	Simple and fast titratio Once daily formulation n Anxiolytic and antidepressant effect	Side effects Drugs interactions
Pregabalin	Lyrica*	No major drugs interactions Anxiolytic effect	Side effects Titration. Cost
Amytriptiline Imipramine Clomipramine	Laroxyl* Tofranil* Anafranil*	Drops Cost Anxiolytic effect (clomipramine) High antidepressant effect if high dose	Titration Anticholinergic and adrenal effects
Oxycodone slow-release Morphine	Oxycontin LP*	Effect on possible inflammatory pain associated	Side effects Addiction
Tramadol		Effect on possible inflammatory pain associated	Side effects Addiction
Carbamazepin	Tegrétol*		Titration: cost Enzyme inducer Side effects
Clonazépam	Rivotril*	Drops Cost	Analgesic effect not studied. Drowsiness,Memory problems Addiction,Withdrawal syndrome

INN = International Nonproprietary Name

## Conclusion

Despite its impact on quality of life and its high prevalence, PDN remains largely under-diagnosed and under-treated. The present study showed that almost 1 in 6 diabetics suffered from PDN, but only 26% received a specific treatment. The availability of a simple screening tool such as DN4 makes its detection and diagnosis easier. Although there is a wide variety of therapeutic options, and many guidelines, none has proven to be satisfactory. Thus, more effective therapeutic combination should be found with limited side effects.

## Competing interests

The authors declare no competing interests.

## Figures and Tables

**Table:** 

		Yes	No
1-	Burning		
2-	Painful cold		
3-	Electrics shocks		

**Table:** 

		Yes	NO
4-	Tingling		
5-	Pins and needles		
6-	Numbless		
7-	Itching		

**Table:** 

8-	Hypoesthesia to touch	Yes	No
9-	Hypoesthesia to prick		
			

**Table:** 

		Yes	No
10	Brushing		

## DN4 questionnaire

Please complete this questionnaire by ticking one answer for each item in the 4 questions below:

## INTERVIEW OF THE PATIENT

**Question 1:** Does the pain have one or more of the following characteristics?

**Question 2:** In the pain associated with one or more of the following symptoms in the same area?

## EXAMINATION OF THE PATIENTS

**Question 3:** Is the pain located in an area where the physical examination may reveal one or more of the following characteristics?

**Question 4:** In the painful area, can the pain be caused or increase by:

## References

[1] International Diabetes Federation (2016). IDF Diabetes Atlas.

[2] Bouhassira D, Letanoux M, Hartemann A (2013). Chronic pain with neuropathic characteristics in diabetic patients: a French cross-sectional study. PLoS One.

[3] Aslam A, Singh J, Rajbhandari S (2014). Pathogenesis of Painful Diabetic Neuropathy. Pain Re-search and treatment.

[4] Hartemann A (2011). Painful diabetic neuropathy: diagnosis and management. Diabetes Metab.

[5] Russell JW, Zilliox LA (2014). Diabetic Neuropathies: continuum. Lifelong learning in neurology.

[6] Dworkin B (2016). The members of the Classification Subcommittee of the Special Interest Group of the International Association for the Study of Pain.

[7] Inoue Y, et Kimura A (1995). Methylglyoxal and regulation of its metabolism in microorganisms. Advances in Microbial Physiology.

[8] Herzberg U, Eliav E, Dorsey JM, Gracely RH, Kopin IJ (1997). NGF involvement in pain induced by chronic constriction injury of the rat sciatic nerve. Neuroreport.

[9] Wu EQ (2007). Estimated prevalence of peripheral neuropathy and associated pain in adults with diabetes in France. Curr Med Res Opin.

[10] Jambart S (2011). Prevalence of painful diabetic peripheral neuropathy among patients with diabetes mellitus in the Middle East region. J Int Med Res.

[11] Aslam A, Singh J, Rajbhandari S (2015). Prevalence of Painful Diabetic Neuropathy Using the Self-Completed Leeds Assessment of Neuropathic Symptoms and Signs Questionnaire in a Population with Diabetes. Can J Diabetes.

[12] Davies M, Brophy S, Williams R, Taylor A (2006). The prevalence, severity, and impact of pain-ful diabetic peripheral neuropathy in type 2 diabetes. Diabetes Care.

[13] Abbott CA, Malik RA, van Ross ER, Kulkarni J, Boulton AJ (2011). Prevalence and characteris-tics of painful diabetic neuropathy in a large community-based diabetic population in the UK. Diabetes Care.

[14] Sui-Whi J, Ming-Shyan L, Wen-Nan C, Beaton R, Mei-Yen C (2016). Prevalence, discomfort and self-relief behaviours of painful diabetic neuropathy in Taiwan: a cross-sectional study. BMJ Open.

[15] Papanas N, Ziegler D (2015). Risk Factors and Comorbidities in Diabetic Neuropathy: an Up-date 20. Rev Diabet Stud.

[16] Ma WY (2012). Variability in hemoglobin A1c predicts all-cause mortality in patients with type 2 diabetes. J Diabetes Complications.

[17] Aouiche S (2014). Neuropathie diabétique douloureuse: fréquence, facteurs de risque et gravité dans une cohorte de 400 sujets diabétiques en Algérie. Médecine des maladies Mé-taboliques.

[18] Partanen J (1995). Natural history of peripheral neuropathy in patients with non-insulin-dependent diabetes mellitus. N Engl J Med.

[19] Wifffen PJ (2012). Enhanced glucose control for preventing and treating diabetic neuropathy. Journal of pain & palliative care pharmacotherapy.

[20] Boussageon R (2011). Effect of intensive glucose lowering treatment on all causes mortality, cardiovascular death, and microvascular events in type 2 diabetes: meta-analysis of randomized controlled trials. BMJ.

[21] Hoeldtke RD, Bryner KD, Van Dyke K (2011). Oxidative stress and autonomic nerve function in early type 1 diabetes. Clin Auton Res.

[22] Low SK (2015). Prevalence of chronic kidney disease in adults with type 2 diabetes mellitus. Ann Acad Med Singapore.

[23] Papanas N, Liakopoulos V, Maltezos E, Stefanidis I (2007). The diabetic foot in end stage renal disease. Ren Fail.

[24] Agrawal RP (2014). Prevalence of micro and macrovascular complications and their risk factors in type-2 diabetes mellitus. J Assoc Physicians India.

[25] Ziegler D (2009). Neuropathic pain in diabetes, prediabetes and normal glucose toler-ance: the MONICA/KORA Augsburg Surveys S2 and S3. Pain Med.

[26] Ziegler D (2009). Prevalence and risk factors of neuropathic pain in survivors of myocar-dial infarction with prediabetes and diabetes: the KORA Myocardial Infarction Registry. Eur J Pain.

[27] Ziegler D (2008). Prevalence of polyneuropathy in pre-diabetes and diabetes is associat-ed with abdominal obesity and macroangiopathy: the MONICA/KORA Augsburg Surveys S2 and S3. Diabetes Care.

[28] Bouhassira D (2012). Douleurs neuropathiques. Arnette.

[29] Bennett MI (2007). Using screening tools to identify neuropathic pain. Pain.

[30] Bouhassira D, Attal N (2011). Diagnosis and assessment of neuropathic paIn: the saga of clin-ical tools. Pain.

[31] Bouhassira D (2005). Comparison of pain syndromes associated with nervous or somatic lesions and development of a new neuropathic pain diagnostic questionnaire (DN4). Pain.

[32] Harifi G (2011). Validity and reliability of the Arabic adapted version of the DN4 ques-tionnaire (Douleur Neuropathique 4 Questions) for differential diagnosis of pain syndromes with a neuropathic or somatic component. Pain Pract.

[33] Daousi C (2004). Chronic painful peripheral neuropathy in an urban community: a con-trolled comparison of people with and without diabetes. Diabet Med.

[34] Javed S, Petropoulos I, Alam U, et Malik R (2015). Treatment of painful diabetic neuropathy. Ther Adv Chronic Dis.

[35] Cotter M, Ekberg K, Wahren J, Cameron N (2003). Effects of proinsulin c-peptide in experi-mental diabetic neuropathy: vascular actions and modulation by nitric oxide synthase inhi-bition. Diabetes.

[36] Spallone V (2012). Management of painful diabetic neuropathy: guideline guidance or jungle?. Curr Diab Rep.

[37] Arezzo JC, Rosenstock J, Lamoreaux L, Pauer L (2008). Efficacy and safety of pregabalin 600 mg/d for treating painful diabetic peripheral neuropathy: a double-blind placebo controlled trial. BMC Neurology.

[38] Richter RW (2005). Relief of painful diabetic peripheral neuropathy with pregabalIn: a randomized, placebo-controlled trial. The journal of pain.

